# Association between oxidative balance score and serum cobalt level in population with metal implants: a cross-sectional study from NHANES 2015–2020

**DOI:** 10.3389/fnut.2024.1485428

**Published:** 2024-12-06

**Authors:** Wenxiu Yuan, Jing Chen, Jun Sun, Chenyang Song, Zhi Chen

**Affiliations:** ^1^Postdoctoral Workstation, Fujian Key Laboratory of Oral Diseases, Fujian Provincial Engineering Research Center of Oral Biomaterial, Stomatological Key Lab of Fujian College and University, Department of Orthodontics, School and Hospital of Stomatology, Fujian Medical University, Fuzhou, China; ^2^Department of Ophthalmology, Fujian Provincial Hospital, Fuzhou, Fujian, China; ^3^Department of Emergency, Zhaotong Traditional Chinese Medicine Hospital, Zhaotong, Yunnan, China; ^4^Department of Orthopedics, Fujian Medical University Union Hospital, Fuzhou, Fujian, China

**Keywords:** metal implant, oxidative balance score, cobalt, NHANES, dietary

## Abstract

**Background:**

Growing evidence indicates that metal implants influence the body’s oxidative stress status, which in turn affects the degradation and stability of metal implants. The oxidative balance score (OBS) is a composite indicator, reflecting the overall oxidative balance of pro-and antioxidants of the human body. However, the associations between OBS and the level of metal ions on the population with metal implants remain to be elucidated.

**Methods:**

We conducted a cross-sectional study using data from 2015 to 2020 National Health and Nutrition Examination Survey (NHANES). Dietary and lifestyle factors closely associated with oxidative stress were quantified to calculate the OBS. Weighted multivariate logistic regression and smooth curve fittings were performed to examine the relationship between OBS and serum cobalt levels. Subgroup analyses were stratified by age and gender. In cases where non-linearity was detected, threshold effects were assessed using a two-piecewise linear regression model.

**Results:**

A total of 549 participants were included in this analysis. The dietary OBS was negatively associated with serum cobalt level in fully adjusted model (*β* = −0.179, 95%CI: −0.358 to −0.001, *P*: 0.04918). Stratified by age and gender, negative correlation of OBS and dietary OBS with serum cobalt level was observed only in men and age over 70 years participants. Threshold effect analysis showed linear relationships between OBS, dietary OBS and cobalt level in males. There were non-linear relationships between OBS, dietary OBS and cobalt level in age over 70 years participants, with inflection points identified at 16.3 and 8.7 for OBS and dietary OBS, respectively.

**Conclusion:**

Our study confirms the inverse relationships between oxidative stress and serum cobalt level in individuals with metal implants, highlighting the significance of optimizing OBS to mitigate the risk of metal ion toxicity. These findings emphasize the importance of maintaining an antioxidant diet and lifestyle, particularly as they offer greater protective effect for males and the elderly population.

## 1 Introduction

Currently, the trend of global aging is becoming increasingly prominent due to persistently low birth rates and extended life expectancy. By 2050, it is projected that the population aged 65 and older will account for 20% of the global population (1), which suggests a significant rise in the incidence of degenerative diseases and related complications, such as osteoporosis, fractures, osteoarthritis (2). In patients with orthopedic diseases, the use of metal implants for fixation or replacement to relieve pain, correct deformities, and restore function has increased annually (2–6). It has been reported that over one million total hip and knee replacement are performed annually in the United States, with the cost exceeding $25 billion (7). However, the long-term survival rate of metal implants is not optimistic. Among younger patients undergoing total hip replacement, only 72% of the implants are able to last for 10 years (8). One of the challenges in the application of metal implants is the generation of metal debris and the release of metal ions. As is well known, these debris and ions can trigger localized adverse reactions, leading to the loosening and failure of the implants, and they may even enter the circulatory system, resulting in systemic damage (9). Numerous studies suggest that the accumulation of metal debris and ions can induce the formation of local pseudotumors (10, 11). Research conducted by Grammatopoulos et al. (12) found that out of 53 cases of metal-on-metal hip replacements, 16 required revision surgery due to the presence of pseudotumors. A prospective study carried out by British researchers revealed that, compared to preoperative values, patients who underwent metal-on-metal hip replacement experienced a significant increase in the incidence of chromosomal aneuploidy and translocations in peripheral blood, with rates rising 2-fold and 1.5-fold, respectively, within 2 years post-surgery (13). As our understanding of the adverse reactions caused by metal implants deepens, the prevention of these adverse effects has gradually become a focal point of research.

An increasing number of studies indicate that the integration process of metal implants with surrounding tissues may trigger a series of physiological and pathological changes (14). In the initial stage of implantation, the interaction between immune cells and the metal materials leads to the activation and secretion of various mediators, such as superoxide anions and hydroxyl radicals (15, 16). Additionally, metal particles generated due to fatigue, fretting, or corrosion can similarly stimulate local cells to produce excessive reactive oxygen species (ROS) (17–21). These intracellular and extracellular ROS may induce local inflammation and alter the chemical environment of the implants, thereby accelerating the degradation of metal implants and the release of metal ions. Metal micro-particles and ions not only cause localized harm but can also penetrate the bloodstream and lymphatic system, spreading throughout various tissues and organs, triggering systemic inflammatory responses and activating the immune system, resulting in tissue damage and functional impairment (22). Although existing research suggests that ROS are an important factor in the reduced stability of metal implants (5), there is currently a lack of reliable indicators to reflect the oxidative state after metal implantation and to elucidate the relationship between oxidative stress and the dissociation of metal ions.

The OBS is a comprehensive metric designed to assess the balance between oxidative stress and antioxidant capacity within the body (23–25). This indicator has been used to identify individuals at high risk for various chronic diseases, such as cardiovascular diseases, diabetes, and cancer, and to implement corresponding intervention measures. Additionally, it is considered an important monitoring indicator for evaluating treatment efficacy (24, 26–29). However, it remains unclear whether the oxidative stress status of patients with metal implants can be adequately assessed using the OBS, and whether this metric can effectively illustrate the relationship between oxidative stress and the dissociation of metal ions. Herein, we conducted a cross-sectional study to investigate the association between OBS and metal ion of the patients with metal implants using a large-scale, community population-based data from NHANES 2015–2020.

## 2 Materials and methods

### 2.1 Data source

The NHANES is a program in the United States designed to assess the health and nutritional status of adults and children. It involves a complex and comprehensive set of data methods, including physical examinations, laboratory tests, and questionnaires. The survey aims to monitor trends in various health indicators, such as diseases, nutritional deficiencies, and exposure to environmental contaminants. The National Center for Health Statistics Ethics Review Board has approved NHANES protocols, with all participants provided consenting to their data’s use in research (30).

### 2.2 Study population

**Figure 1:**
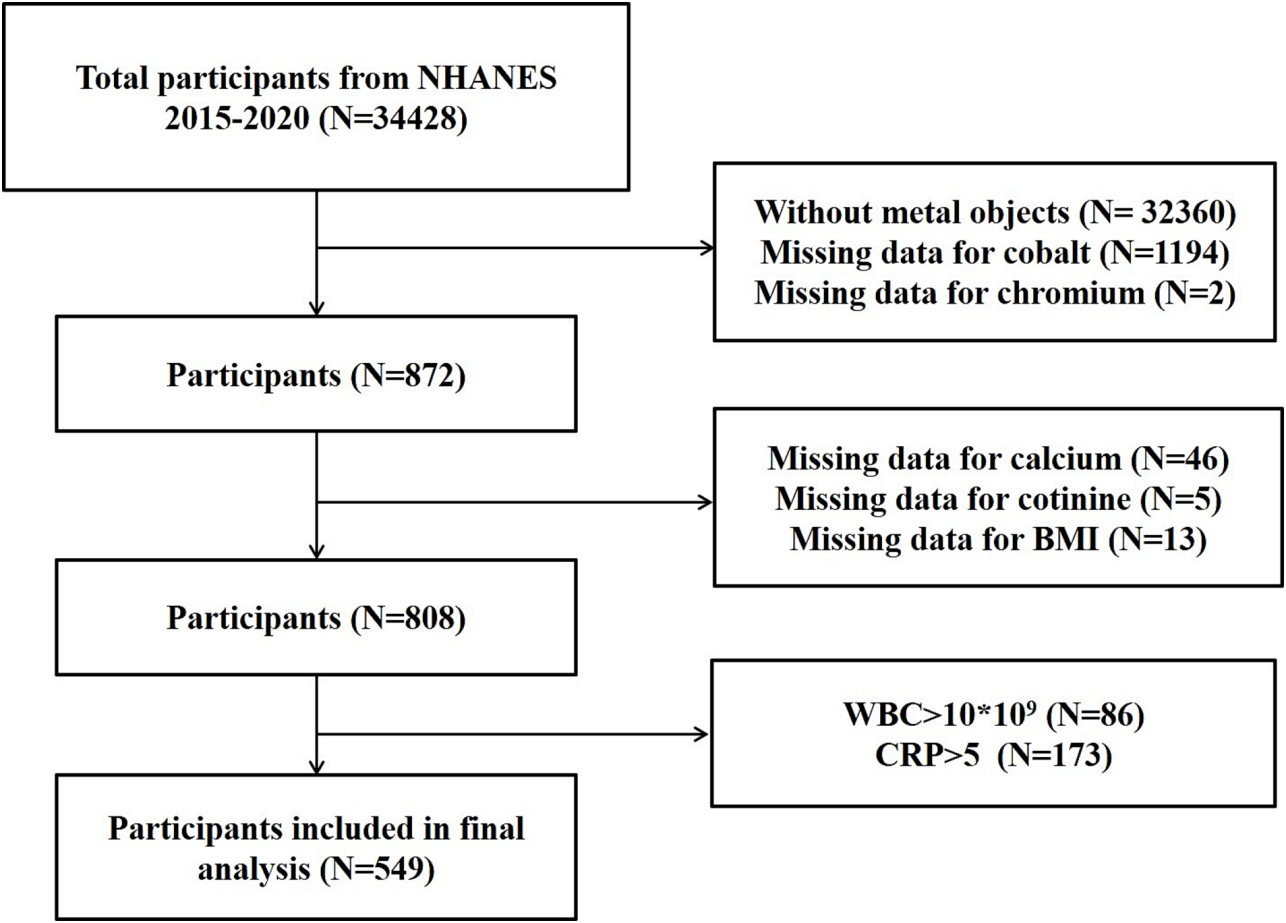
Flowchart diagram depicting the selection strategy of study participants.

In this study, we analyzed NHANES data from three consecutive 2-year cycles spanning 2015–2020. We included participants with metal objects inside body, and had complete data of OBS components, serum cobalt, and serum chromium. We excluded participants without metal objects (*n* = 32,360), with missing data for cobalt (*n* = 1,194), chromium (*n* = 2), calcium (*n* = 46), cotinine (*n* = 5), Body mass index (BMI) (*n* = 13), with indication of infection (WBC > 10 × 10^9^/L; *n* = 86) and inflammation (CRP > 5 mg/L; *n* = 173). The participant screening processes is presented in Figure 1.

For each participant enrolled, data on OBS components, serum cobalt, and serum chromium, and covariates were extracted and analyzed.

### 2.3 Study variables

#### 2.3.1 Independent variables

Based on prior research about the relationship of nutrients and lifestyle factors with oxidative stress, 16 nutrients and three lifestyle factors (alcohol consumption, smoking, and BMI) were collected to calculate OBS, including five pro-oxidants and 14 antioxidants (31, 32). Among the variables assessed, 16 nutrients and alcohol were derived from the mean of the various ingredients from the dietary interview on first day to determine the quantiles thresholds for scoring purposes. Smoking was estimated by serum cotinine, which was measured by an isotope dilution-high performance liquid chromatography/atmospheric pressure chemical ionization tandem mass spectrometry (ID HPLC-APCI MS/MS). The BMI (kg/m^2^) was collected from body measures and calculated as weight in kilograms divided by height in meters square. We stratified all components into three distinct groups, corresponding to the first, second, and third tertiles, respectively. Antioxidants were allocated fractional values ranging from 0 to 2, whereas the scoring for pro-oxidants were inversely distributed. Finally, the OBS scores for each participant were aggregated to yield the individual final OBS values. Supplementary Table S1 shows the distribution scheme of OBS components.

#### 2.3.2 Dependent variables

Whole blood specimens were processed, stored, and shipped to the Division of Laboratory Sciences, National Center for Environmental Health, Centers for Disease Control and Prevention, Atlanta, GA for analysis. The concentrations of cobalt (nmol/L) and chromium (nmol/L) in whole blood specimens were directly measured using inductively coupled plasma mass spectrometry (ICP-MS) (33).

#### 2.3.3 Covariates

All covariates were identified based on findings from previous studies (22, 34). The demographic data, including age (year), gender (male/female), and ethnicity (Hispanic, non-Hispanic White, non-Hispanic Black, and Other Races), were collected. BMI was obtained from body measures. The criteria for identifying hypertension and diabetes were based on participants’ self-reported medical diagnoses validated by a physician. Smoking status was ascertained through Questionnaire Data. Participants who had smoked fewer than 100 lifetime cigarettes were categorized as never smokers. Those who had smoked at least 100 cigarettes, but were not smoking at the time of the survey were classified as former smokers. Conversely, participants who had exceeded the 100 cigarette threshold and were actively smoking at the time of the survey were identified as current smokers (35). The neutrophil count (1,000 cells/μL), neutrophil percentage (%), lymphocyte count (1,000 cells/μL), monocyte number (1,000 cells/μL), and platelet count (1,000 cells/μL), were quantified utilizing the Complete Blood Count with a Five-Part Differential methodology. Blood urea nitrogen (mg/dL), iron (ug/dL), phosphorus (mg/dL), total protein (g/dL), albumin (g/dL), and uric acid (mg/dL) were assessed using Beckman UniCel^®^ DxC800 Synchron. HDL-cholesterol (mg/dL) and total cholesterol (mg/dL) were quantified using Roche/Hitachi Modular P Chemistry Analyzer. The albumin in urine (mg/dL) was measured using a solid-phase fluorescent immunoassay by sequoia-Turner Digital Fluorometer, Model 450, urine creatinine (mg/dL) was determined by Enzymatic using Roche Cobas 6,000 Analyzer. Urinary albumin-creatinine ratio (UACR) was calculated by dividing urinary albumin by creatinine (36). Monocyte/HDL cholesterol ratio (MHR) was calculated as monocyte number divided by HDL cholesterol (37). Systemic immune-inflammation indicator (SII) was calculated by multiplying the platelet count by the neutrophil count and dividing by the lymphocyte count (38). Neutrophil percentage to albumin ratio (NPAR) was calculated as neutrophil percentage divided by albumin (39). The NHANES website provides full information on the laboratory procedures, data processing, quality control, and analytic notes.

### 2.4 Statistical analysis

The statistical software packages R 3.4.3^1^ and EmpowerStats 2.0^2^ were used to conduct data analysis, with *p* < 0.05 was considered statistically significant. All estimates were calculated with consideration of the NHANES sample weights. Continuous variables were compared using a weighted linear regression model, while categorical variables were assessed with a weighted chi-square test. Weighted multivariable linear regression analyses were performed to investigate the relationship of OBS with serum cobalt and chromium. Further investigation was conducted through subgroup analyses, stratified by age and gender. The presence of non-linear relationships was examined using generalized additive models and smooth curve fittings. When non-linearity was detected, a two-piecewise linear regression model was employed to analyze the threshold effect.

## 3 Results

### 3.1 Baseline characteristics

In accordance with the inclusion and exclusion criteria, a total of 549 participants were deemed eligible for inclusion in the definitive analysis. These participants were divided into four groups based on OBS levels. As the levels of OBS escalate, there is a progressive decline in the levels of serum cobalt, BMI, urine creatinine, and uric acid, as well as the incidence rates of diabetes and hypertension (Table 1).

**Table 1:** Baseline characteristics of study participants.

	OBS				
Characteristics	<10	≥10, <20	≥20, <30	≥30	*p-*value
*n*	66	203	225	55	
Age (years)	62.292 ± 12.112	64.521 ± 10.159	61.054 ± 12.267	61.236 ± 12.192	0.01531
BMI (kg/m^2^)	31.124 ± 5.723	29.693 ± 5.075	29.545 ± 6.778	25.773 ± 3.402	<0.00001
Chromium (nmol/L)	9.512 ± 16.489	10.324 ± 9.739	8.210 ± 7.720	8.613 ± 7.542	0.13219
Cobalt (nmol/L)	10.269 ± 40.897	3.460 ± 7.514	4.370 ± 8.265	2.787 ± 1.290	0.01864
Urine creatinine (mg/dL)	122.523 ± 91.860	103.849 ± 62.542	98.079 ± 64.584	75.665 ± 48.977	0.00152
Blood urea nitrogen (mg/dL)	15.699 ± 7.220	16.286 ± 5.444	16.433 ± 4.857	16.791 ± 4.212	0.73797
Iron (μg/dL)	89.411 ± 26.580	85.607 ± 28.057	86.114 ± 33.064	86.230 ± 37.065	0.91010
Phosphorus (mg/dL)	3.673 ± 0.514	3.666 ± 0.494	3.709 ± 0.564	3.728 ± 0.514	0.77810
Total Protein (g/dL)	7.049 ± 0.413	6.971 ± 0.407	6.910 ± 0.361	7.055 ± 0.328	0.01160
Uric acid (mg/dL)	5.375 ± 1.360	5.591 ± 1.361	5.336 ± 1.362	4.750 ± 1.038	0.00016
Monocyte number (1,000 cells/μL)	0.626 ± 0.187	0.571 ± 0.163	0.574 ± 0.178	0.596 ± 0.298	0.31775
HDL-cholesterol (mg/dL)	55.236 ± 16.203	57.743 ± 22.547	56.273 ± 19.857	60.625 ± 16.961	0.39335
Total cholesterol (mg/dL)	186.738 ± 32.688	192.013 ± 43.755	193.473 ± 47.404	198.341 ± 32.718	0.55408
MHR	0.012 ± 0.005	0.012 ± 0.007	0.012 ± 0.006	0.011 ± 0.007	0.66601
UACR	0.520 ± 2.228	0.208 ± 1.025	0.559 ± 2.937	0.240 ± 0.622	0.34465
NPAR	0.949 ± 0.298	0.904 ± 0.262	0.951 ± 0.285	0.835 ± 0.283	0.01519
SII	464.666 ± 210.373	483.256 ± 250.736	520.514 ± 255.919	501.649 ± 215.223	0.33337
Dietary OBS	4.371 ± 1.365	11.350 ± 2.756	20.929 ± 3.131	26.585 ± 1.436	<0.00001
Lifestyle OBS	3.077 ± 1.120	3.397 ± 1.302	3.552 ± 1.349	4.979 ± 1.083	<0.00001
Gender					0.50096
Male	47.250	43.238	47.368	37.636	
Female	52.750	56.762	52.632	62.364	
Race					0.11863
Hispanic	9.240	9.561	7.862	4.006	
Non-Hispanic White	70.385	74.423	79.801	85.374	
Non-Hispanic Black	16.173	6.800	4.519	3.096	
Other	4.202	9.215	7.817	7.524	
Diabetes					0.02921
Yes	24.417	19.821	12.504	5.818	
No	74.145	77.318	85.133	93.695	
Borderline	1.438	2.861	2.364	0.487	
Hypertension					0.00271
Yes	68.423	53.094	45.113	36.083	
No	31.577	46.906	54.887	63.917	
Smoking					0.28010
Never	37.659	39.608	47.766	48.476	
Former	37.057	41.651	36.792	41.180	
Still	25.283	18.742	15.442	10.343	

MHR, monocyte/high density lipoprotein cholesterol ratio; UACR, urinary albumin/creatinine ratio; NPAR, neutrophil percentage to albumin ratio; SII, systemic immune-inflammation indicator. Mean ± SD for continuous variables: *P-*value was calculated by weighted linear regression model. Percent for categorical variables: *P-*value was calculated by weighted chi-square test.

### 3.2 Association between OBS, dietary OBS, lifestyle OBS, and serum cobalt

Multiple linear regression analyses were conducted to evaluate the correlations between OBS, dietary OBS, lifestyle OBS, and serum cobalt level. The unadjusted and adjusted outcomes of these analyses are presented in Table 2. In all models examined, no significant correlation was observed between OBS and lifestyle OBS with respect to serum cobalt levels. Dietary OBS was found negatively associated with serum cobalt levels in fully adjusted model (model 3: *β* = −0.179, 95%CI: −0.358 to −0.001, *P*: 0.04918), but not in unadjusted and partially adjusted model.

**Table 2:** Relationships of OBS, dietary OBS, lifestyle OBS, and the cobalt level.

Outcome	Model 1		Model 2		Model 3	
	*β* (95%CI)	*p*-value	*β* (95%CI)	*P*-value	*β* (95%CI)	*P*-value
OBS	−0.100 (−0.256, 0.056)	0.20771	−0.086 (−0.246, 0.075)	0.29628	−0.155 (−0.328, 0.018)	0.07924
Dietary OBS	−0.129 (−0.292, 0.035)	0.12269	−0.109 (−0.278, 0.059)	0.20412	−0.179 (−0.358, −0.001)	0.04918
Lifestyle OBS	0.485 (−0.350, 1.320)	0.25504	0.390 (−0.473, 1.253)	0.37604	0.556 (−0.564, 1.676)	0.33119

Model 1: no covariates were adjusted.

Model 2: age, M-HDL, and UACR were adjusted.

Model 3: age, BMI (kg/m^2^), diabetes, hypertension, smoking, urine creatinine (mg/dL), blood urea nitrogen (mg/dL), iron (μg/dL), phosphorus (mg/dL), total Protein (g/dL), uric acid (mg/dL), monocyte number (1,000 cells/μL), HDL-Cholesterol (mg/dL), total Cholesterol (mg/dL), M-HDL, UACR, NPAR, and SII were adjusted.

### 3.3 Subgroup analysis for the relationship of OBS, dietary OBS, lifestyle OBS, and serum cobalt

When stratified by gender, negative relationships of OBS (model 3: *β* = −0.259, 95%CI: −0.487 to −0.032, *P*: 0.02626) and dietary OBS (model 3: *β* = −0.288, 95%CI: −0.524 to −0.052, *P*: 0.01740) with serum cobalt were found to be statistically significant in males while not in females (Table 3).

**Table 3:** Relationships of OBS, dietary OBS, lifestyle OBS, and the cobalt level stratified by gender.

Outcome	Male		Female	
	*β* (95%CI)	*P*-value	*β* (95%CI)	*P*-value
Model 1				
OBS	−0.153 (−0.356, 0.051)	0.14243	−0.054 (−0.288, 0.180)	0.65016
Dietary OBS	−0.186 (−0.398, 0.027)	0.08746	−0.076 (−0.322, 0.169)	0.54266
Lifestyle OBS	0.479 (−0.600, 1.558)	0.38494	0.442 (−0.822, 1.707)	0.49358
Model 2				
OBS	−0.124 (−0.328, 0.080)	0.23453	−0.046 (−0.289, 0.197)	0.71257
Dietary OBS	−0.154 (−0.368, 0.060)	0.16057	−0.063 (−0.318, 0.192)	0.62932
Lifestyle OBS	0.428 (−0.662, 1.517)	0.44209	0.332 (−0.981, 1.645)	0.62048
Model 3				
OBS	−0.259 (−0.487, −0.032)	0.02626	−0.102 (−0.376, 0.171)	0.46308
Dietary OBS	−0.288 (−0.524, −0.052)	0.01740	−0.122 (−0.403, 0.158)	0.39272
Lifestyle OBS	0.332 (−1.175, 1.839)	0.66641	0.528 (−1.167, 2.222)	0.54214

Model 1: no covariates were adjusted.

Model 2: age, M-HDL, and UACR were adjusted.

Model 3: age, BMI (kg/m^2^), diabetes, hypertension, smoking, creatinine, urine (mg/dL), blood urea nitrogen (mg/dL), iron (μg/dL), phosphorus (mg/dL), total Protein (g/dL), uric acid (mg/dL), monocyte number (1,000 cells/μL), HDL-Cholesterol (mg/dL), total cholesterol (mg/dL), M-HDL, UACR, NPAR, and SII were adjusted.

In subgroup analysis stratified by age, we observed negative relationships of OBS (model 3: *β* = −0.545, 95%CI: −0.982 to −0.108, *P*: 0.01545) and dietary OBS (model 3: *β* = −0.657, 95%CI: −1.110 to −0.205, *P*: 0.00496) with serum cobalt only in ≥70 years old participants, but not in other age groups (Table 4).

**Table 4:** Relationship of OBS, dietary OBS, lifestyle OBS, and the cobalt level stratified by age.

Outcome	<50		≥50, <60		≥60, <70		≥70	
	*β* (95%CI)	*P*-value	*β* (95%CI)	*P*-value	*β* (95%CI)	*P*-value	*β* (95%CI)	*P*-value
Model 1								
OBS	−0.032 (−0.028, 0.091)	0.30203	−0.019 (−0.147, 0.109)	0.77068	0.103 (−0.169, 0.375)	0.46034	−0.332 (−0.738, 0.075)	0.11123
Dietary OBS	0.019 (−0.043, 0.082)	0.54829	−0.031 (−0.168, 0.106)	0.65973	0.136 (−0.136, 0.409)	0.32842	−0.432 (−0.868, 0.004)	0.05367
Lifestyle OBS	0.384 (0.081, 0.688)	0.01522	0.231 (−0.460, 0.923)	0.51312	−1.001 (−2.502, 0.500)	0.19303	1.205 (−0.995, 3.404)	0.28423
Model 2								
OBS	0.026 (−0.032, 0.085)	0.37504	−0.025 (−0.158, 0.108)	0.71546	0.109 (−0.184, 0.403)	0.46614	−0.413 (−0.820, −0.005)	0.04846
Dietary OBS	0.016 (−0.045, 0.076)	0.61676	−0.036 (−0.178, 0.106)	0.61878	0.145 (−0.145, 0.436)	0.32802	−0.509 (−0.942, −0.077)	0.02212
Lifestyle OBS	0.337 (0.038, 0.637)	0.03006	0.201 (−0.516, 0.918)	0.58347	−1.184 (−2.798, 0.431)	0.15292	1.065 (−1.190, 3.321)	0.35565
Model 3								
OBS	0.022 (−0.039, 0.084)	0.48221	−0.098 (−0.266, 0.070)	0.25728	0.216 (−0.125, 0.558)	0.21683	−0.545 (−0.982, −0.108)	0.01545
Dietary OBS	0.019 (−0.043, 0.082)	0.54491	−0.114 (−0.291, 0.063)	0.20996	0.252 (−0.083, 0.586)	0.14332	−0.657 (−1.110, −0.205)	0.00496
Lifestyle OBS	0.144 (−0.257, 0.545)	0.48342	0.172 (−0.843, 1.187)	0.74097	−1.649 (−3.722, 0.424)	0.12152	2.836 (−0.191, 5.862)	0.06798

Model 1: no covariates were adjusted.

Model 2: M-HDL, and UACR were adjusted.

Model 3: BMI (kg/m^2^), diabetes, hypertension, smoking, urine creatinine (mg/dL), blood urea nitrogen (mg/dL), iron (μg/dL), phosphorus (mg/dL), total Protein (g/dL), uric acid (mg/dL), monocyte number (1,000 cells/μL), HDL-Cholesterol (mg/dL), total Cholesterol (mg/dL), M-HDL, UACR, NPAR, and SII were adjusted.

### 3.4 Threshold effect analysis

**Figure 2:**
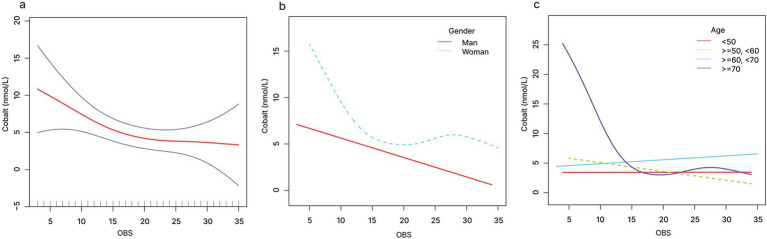
The relationship between OBS and serum cobalt **(A)**, stratified by gender **(B)**, stratified by age **(C)**.

**Figure 3:**
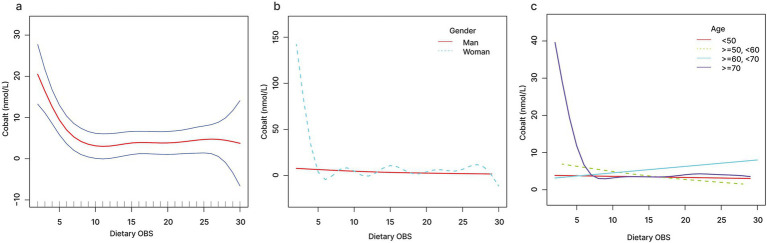
The relationship between dietary OBS and serum cobalt **(A)**, stratified by gender **(B)**, stratified by age **(C)**.

The relationship between OBS and serum cobalt exhibited a non-linear pattern, as shown by smooth curve fitting (Figure 2A). When OBS < 19.7, a one-unit increase in OBS level was associated with 0.5 units decrease in serum cobalt level. When OBS was >19.7, no significant association was observed with serum cobalt (Figure 2A and Table 5). Similarly, dietary OBS and serum cobalt exhibited a non-linear relationship as shown by smooth curve fitting (Figure 3A). When dietary OBS < 9.6, a one-unit increase in dietary OBS was associated with 2.225 units decrease in serum cobalt level. When OBS was >9.6, no significant association was observed with serum cobalt (Figure 3A and Table 5).

**Table 5:** Threshold effect analysis of OBS and dietary OBS on the cobalt level using the two-piecewise linear regression model.

	Adjusted *β* (95%CI)	*P-*value
OBS infection point	19.7	
OBS < 19.7	−0.500 (−0.876, −0.125)	0.0093
OBS > 19.7	0.154 (−0.191, 0.499)	0.3818
Log likelihood ratio	0.039	
Dietary OBS infection point	9.6	
Dietary OBS < 9.6	−2.225 (−3.120, −1.329)	<0.0001
Dietary OBS > 9.6	0.161 (−0.067, 0.389)	0.1662
Log likelihood ratio	<0.001	

The age, BMI (kg/m^2^), diabetes, hypertension, smoking, urine creatinine (mg/dL), blood urea nitrogen (mg/dL), iron (μg/dL), phosphorus (mg/dL), total Protein (g/dL), uric acid (mg/dL), monocyte number (1,000 cells/μL), HDL-Cholesterol (mg/dL), total Cholesterol (mg/dL), M-HDL, UACR, NPAR, and SII were adjusted.

When stratified by gender, linear relationships of OBS and dietary OBS with serum cobalt were detected in males (Figures 2B, 3B). When stratified by age, non-linear relationships of OBS and dietary OBS with serum cobalt were observed in ≥70 years old participants (Figures 2C, 3C). When OBS < 16.3, a one-unit increase in OBS level was associated with 1.904 units decrease in serum cobalt level. When OBS was >16.3, no significant association was observed with serum cobalt. Similarly, when dietary OBS < 8.7, a one-unit increase in dietary OBS level was associated with 5.184 units decrease in serum cobalt level. When dietary OBS was >8.7, no significant association was observed with serum cobalt (Table 6).

**Table 6:** Threshold effect analysis of OBS and dietary OBS on the cobalt level in ≥70 participants using the two-piecewise linear regression model.

	Adjusted *β* (95%CI)	*P-*value
OBS infection point	16.3	
OBS < 16.3	−1.904 (−3.261, −0.548)	0.0066
OBS > 16.3	0.060 (−0.660, 0.780)	0.8711
Log likelihood ratio	0.029	
Dietary OBS infection point	8.7	
Dietary OBS < 8.7	−5.184 (−7.497, −2.871)	<0.0001
Dietary OBS > 8.7	0.053 (−0.510, 0.615)	0.8548
Log likelihood ratio	<0.001	

The age, BMI (kg/m^2^), diabetes, hypertension, smoking, creatinine, urine (mg/dL), blood urea nitrogen (mg/dL), iron (μg/dL), phosphorus (mg/dL), total Protein (g/dL), uric acid (mg/dL), monocyte number (1,000 cells/μL), HDL-Cholesterol (mg/dL), total Cholesterol (mg/dL), M-HDL, UACR, NPAR, and SII were adjusted.

## 4 Discussion

To investigate the association between oxidative stress and metal ion levels in patients with metal implants, we conducted a large-scale cross-sectional study involving 549 representative participants based on data from NHANES 2015–2020. Our results indicated that as the OBS increased, serum cobalt levels gradually decreased. These findings highlight the importance of managing OBS in individuals with metal implants. Elevated OBS and dietary OBS levels are indicative of lower cobalt levels, ultimately helping to minimize the impact of metal ions on health.

Aging is an irreversible trend that has led to a significant increase in orthopedic degenerative diseases and their complications among the elderly population, thereby resulting in a continuous rise in the demand for metal implants. Epidemiological studies indicate that the prevalence of chronic diseases, such as hypertension, coronary heart disease, and diabetes, is relatively high in this demographic. Therefore, it is crucial to explore the factors influencing the progression of chronic disease in patients who have undergone metal implantation. Oxidative stress refers to the imbalance between antioxidant defense mechanisms and the production of ROS, and it has been confirmed to be closely associated with various chronic diseases. Oxidative stress is considered a significant contributing factor to cardiovascular diseases like atherosclerosis, heart disease, and hypertension, and it also plays a vital role in the onset and progression of diabetes (40). Hyperglycemic states can trigger the production of ROS, leading to insulin resistance and *β*-cell dysfunction. Furthermore, oxidative stress is closely related to complications of diabetes, including cardiovascular diseases, kidney disease, and neuropathy (41). Our research has revealed that as OBS levels increase, the incidence of diabetes and hypertension gradually decreases, and renal function also shows improvement. This finding suggests that maintaining a healthy lifestyle and a diet rich in antioxidants can help mitigate the progression of chronic diseases in patients receiving metal implant therapy.

Mechanical wear and fluid corrosion have long been recognized as the primary factors leading to the generation of metallic particles and the release of metal ions (42, 43). In recent years, the role of oxidative stress in this process has garnered increasing attention. Research indicates that oxidative stress exacerbates the generation of metallic particles and the release of metal ions through various mechanisms, such as enhancing the corrosion rate of metal implants, promoting inflammatory responses, intensifying cellular damage and apoptosis, and altering the surface characteristics of materials. The increase in ROS accelerates the degradation of implants while diminishing the tissue’s ability to clear metal ions, thereby exacerbating the accumulation of metallic particles and ions in the surrounding environment (44–47). Xu et al. (44) observed extensive infiltration and accumulation of macrophages in the synovial tissue of patients with failed metal hip prostheses. Their study revealed that cobalt released from the metal implants stimulate surrounding immune cells to produce ROS, which subsequently downregulate the RhoA signaling pathway in macrophages. This alteration results in increased formation of intracellular podosome-type adhesion structures and enhanced adhesion to the extracellular matrix, ultimately leading to decreased motility of the macrophages (44). Furthermore, Kim et al. (47) found that ROS induced by metal implants could trigger apoptosis in osteoblasts and gingival fibroblasts through the activation of the Nrf2/ARE pathway and the upregulation of heme oxygenase-1. What is even worse, the significant entry of metal ions into the bloodstream can provoke systemic toxic reactions, impacting the functionality of vital organs. Metals such as cobalt, chromium, nickel, and titanium, which are major components of metal implants, have gained increasing attention due to the health issues they may cause. In a prospective study involving 100 patients with metal implants, researcher Brodner evaluated the serum cobalt concentrations in patients following metal-on-metal total hip arthroplasty, finding that the serum cobalt levels exceeded the detection limit (48). Another study described a 70-year-old patient with cobalt toxicity, whose primary symptoms included progressive hearing and vision deterioration, cataracts, and axonal sensorimotor neuropathy (49). Signorello et al. (50) found that cobalt released from metal implants enters the bloodstream and ultimately accumulates in large amounts in the bladder, resulting in a significant increase in the incidence rate of bladder cancer among patients undergoing hip replacement surgeries. Building on this, Speer et al. (46) investigated the effects of cobalt on human urothelial cells and revealed that soluble cobalt induces cell cycle arrest, leading to cytotoxicity and genotoxicity. Moreover, elevated levels of serum cobalt have also been found to closely associated with increased risks of cardiovascular diseases, hormonal imbalances, immune system suppression, and reduced capacity for infection resistance (21, 51, 52). In this study, we observed a negative correlation between OBS, dietary OBS, and the serum cobalt levels in patients with metal implant. When OBS is less than 19.7, for every unit increase in OBS, the cobalt level decreased by 0.5 units. Similarly, when dietary OBS is less than 9.6, the cobalt levels reduced by 2.225 units as the unit dietary OBS raised. This suggests that maintaining a healthy lifestyle and dietary habits may be an effective strategy to reduce ion dissociation from metal implants, lower related complications, and improve the long-term survival rate of implants.

Previous studies have indicated that there are significant differences in the response to oxidative stress based on gender and age. Before puberty, girls appear to be more susceptible to metabolic dysfunction induced by oxidative stress, whereas elevated redox markers in boys seem to offer protection against arterial stiffness and maintain lipid homeostasis (53). This phenomenon suggests that sex may play a crucial role in regulating oxidative stress-related genes, such as NCF2 and NOX3. Furthermore, research has demonstrated that sex hormones can influence the expression and activity of NADPH oxidase genes and myeloperoxidase, resulting in differences in the response to oxidative stress between males and females (54). Antioxidant lifestyles have been shown to play a significant protective role in the prevention and treatment of depression in women (23). Additionally, Cao’s research has found stronger protective effects of dietary antioxidants in women, suggesting that dietary changes can effectively prevent chronic kidney disease (55). This phenomenon may be attributed to the regulation of most proteins involved in redox status and mitochondrial function by sex hormones (56). The expression of mitochondrial related genes has been shown to be closely related to gender, suggesting a key role of sex hormone signaling in mitochondrial dynamics and cellular redox biology (57). When activated, estrogen-related receptors improve fatty acid oxidation, mitochondrial dynamics, and respiratory chain activity (58, 59). The results of our study indicate that the negative correlation between OBS and serum cobalt levels is pronounced in men but not as evident in women. This discrepancy may be related to differences in hormone levels, as estrogen is known to combat oxidative stress. Itagaki et al. (60) have found that estradiol inhibits the production of ROS and MAPK signaling, thereby preventing the activation of transcription factors and inactivating the downstream transcription processes involved in the expression and activation of TGF-*β*. Additionally, Sun’s research indicates that *β*-estradiol can enhance ROS generation and RUBICON expression, further promoting LC3B-associated phagocytosis in macrophages, which suggests a novel perspective for understanding the mechanism of trained immunity in gender differences during sepsis response (61). Furthermore, women tend to adopt healthier lifestyle choices compared to men, which may contribute to a lack of significant impact on their serum cobalt levels. Consequently, dietary modifications may be more beneficial for lowering serum cobalt levels in men.

Moreover, subgroup analyses reveal significant differences in the association between OBS and serum cobalt levels across various age groups. A notable negative correlation is observed in participants over the age of 70, while this correlation is less pronounced in other age groups. This finding is consistent with previous research by Qu, which highlighted a stronger negative correlation between OBS and periodontitis in the elderly (24). Xiao et al. (62) comprehensively identified redox-modified disease networks that are remodeled in aged mice, establishing a systemic molecular foundation for the complex links between redox dysregulation and tissue aging. They found that the bladders of aged mice exhibit baseline reactive oxygen species (ROS) accumulation and heightened oxidative stress. Mysorekar’ team discovered that d-mannose treatment reversed autophagy flux, rescued the senescence-associated secretory phenotype, and alleviated ROS and the shedding of NLRP3/Gasdermin/IL-1β-driven pyroptotic epithelial cell in elderly animals (63). These phenomena may be associated with the decline in cellular repair capacity and the efficiency of antioxidant defense systems that often accompany aging. Therefore, it is crucial for older adults to improve their OBS in order to reduce serum cobalt levels and mitigate the toxic effects of cobalt.

This study exhibits prominent strengths. Firstly, the data from NHANES were obtained by a sophisticated, multi-stage probability sampling design, strictly adhered to comprehensive quality control to ensure the effectiveness and integrity of the dataset. Therefore, based on the database, our results are highly credible when extended to non-institutionalized populations, especially, the association was validated to be robust after the adjustment for various confounders. Second, an increasing number of studies have demonstrated a significant correlation between CRP levels and biomarkers associated with oxidative stress, indicating that inflammation and infection may be potential factors influencing oxidative stress. To mitigate this impact, we implemented stringent exclusion criteria to omit individuals with CRP levels exceeding 5 mg/L, thereby enhancing the reliability of our findings (64, 65). Third, the present study concentrated on the OBS, encompassing a composite indicator of antioxidant lifestyle and diet, rather than monitoring single component in isolation. This approach enables a more thoroughgoing understanding of the intricate interplay among diverse diet and lifestyle factors in the population with metal implants, as well as their association with the serum cobalt level. Fourth, our study found the association between OBS and serum cobalt level for the first time in population with metal implants and uncovered the gender-specific and age-specific effects of OBS on the serum cobalt level. Fifth, the use of an appropriate covariate adjustment increased the representativeness and reliability of our study. Therefore, the findings carry vital public health implications in the mitigation of the metal ions toxicity on human with metal implants.

Nevertheless, there were also a few limitations in this study. Firstly, the grading criteria for physical activity lack uniformity and certain essential data are inaccessible. Therefore, physical activity was excluded from the calculation of the OBS score. Second, our design of a cross-sectional survey restricts causal inferences between OBS and serum cobalt level. So further prospectively designed studies are needed to verify the causality between OBS and serum cobalt level in human with metal objects. Meanwhile, the biases of recall and reporting using self-reported questionnaires may compromise the accuracy of OBS calculations. Third, the level of metal ions in the human body is influenced by environment and occupation. However, due to privacy concerns, the NHANES database fail to get the geographical location and living status of participants, which makes it impossible to estimate the impact of environmental and occupational exposure on metal ions of human body. Moreover, the uncertainty of implantation type, quantity, reason, or duration, determined the highly heterogeneity of the included population. Finally, the level of metal ions may alter with prolonged postoperative time, so a longer follow-up is extremely needed to further clarify the relationship between the OBS and the serum cobalt level in the population with metal implants.

## 5 Conclusion

In conclusion, data from a nationally representative sample uncovers the significant negative association between OBS and the level of serum cobalt in the population with metal implants. This negative correlation has been corroborated across different gender and age subgroups. Notably, the protective effects of an antioxidant diet and healthy lifestyle are particularly pronounced among males and individuals aged over 70. For the population with metal implants, maintaining good dietary and lifestyle habits may help reduce the generation of metal particles and the dissociation of metal ions, thereby improving the survival rate of metal implants and decreasing the risk of related complications.

## Data Availability

The original contributions presented in the study are included in the article/Supplementary material, further inquiries can be directed to the corresponding author.

## Footnotes

^1^ https://www.r-project.org/

^2^ http://www.empowerstats.com
