# Supplementary material for: Association between oxidative balance score and serum cobalt level in population with metal implants: a cross-sectional study from NHANES 2015–2020
Source: Front Nutr. 2024 Dec 6;11:1485428. doi: 10.3389/fnut.2024.1485428 (PMC11659013; doi:10.3389/fnut.2024.1485428)
Supplement: Supplementary file 1 [file Table_1.docx]

**Supplemental table 1. Oxidative balance score assignment scheme**

| **OBS components** | **Property** | **Male** | | | | **Female** | | |
| --- | --- | --- | --- | --- | --- | --- | --- | --- |
| **Dietary OBS components** | | 0 | 1 | | 2 | 0 | 1 | 2 |
| Calcium (mg/d) | A | <691 | | 692-1148 | ≥1149 | <563 | 564-910 | ≥911 |
| Magnesium (mg/d) | A | <251 | | 252-371 | ≥372 | <201 | 202-301 | ≥302 |
| Zinc (mg/d) | A | <8.74 | | 8.75-13.71 | ≥13.72 | <6.55 | 6.56-10.1 | ≥10.1 |
| Copper (mg/d) | A | <0.944 | | 0.945-1.39 | ≥1.391 | <0.834 | 0.835-1.199 | ≥1.2 |
| Selenium (mcg/d) | A | <90.7 | | 90.8-137.3 | ≥137.4 | <68.8 | 68.9-103.5 | ≥103.6 |
| Dietary fiber (g/d) | A | <12.4 | | 12.5-21.7 | ≥21.8 | <11.2 | 11.3-17.8 | ≥17.9 |
| Carotene (RE/d) | A | <509 | | 509.1-939.75 | ≥939.76 | <417.17 | 417.18-792.83 | ≥792.84 |
| Riboflavin (mg/d) | A | <1.634 | | 1.635-2.516 | ≥2.517 | <1.272 | 1.273-2.014 | ≥2.015 |
| Niacin (mg/d) | A | <20.401 | | 20.402-29.636 | ≥29.637 | <14.448 | 14.449-21.769 | ≥21.77 |
| Vitamin B6 (mg/d) | A | <1.618 | | 1.619-2.439 | ≥2.44 | <1.064 | 1.065-1.834 | ≥1.835 |
| Total folate (mcg/d) | A | <312 | | 313-461 | ≥462 | <234 | 235-369 | ≥370 |
| Vitamin B12 (mcg/d) | A | <2.97 | | 2.98-5.53 | ≥5.54 | <2.12 | 2.13-4.3 | ≥4.31 |
| Vitamin C (mg/d) | A | <33.8 | | 33.9-104.5 | ≥104.6 | <29.4 | 29.5-80.9 | ≥81 |
| Vitamin E (mg/d) | A | <5.93 | | 5.94-10.58 | ≥10.59 | <5.13 | 5.14-8.97 | ≥8.98 |
| Total fat (g/d) | P | ≥101.79 | | 69.73-101.78 | <65.97 | ≥80.85 | 54.04-80.84 | <54.03 |
| Iron (mg/d) | P | ≥17.27 | | 11.46-17.26 | <11.45 | ≥13.65 | 8.69-13.64 | ≤8.68 |
| **Lifestyle OBS components** | | | | | | | | |
| Alcohol (g/d) | P | ≥11.2 | | 0-11.2 | 0 | ≥15 | 0-15 | 0 |
| BMI | P | ≥30.9 | | 26.6-30.8 | <26.5 | ≥30.8 | 25.6-30.7 | <25.6 |
| Cotinine (ng/mL) | P | ≥0.078 | | 0.011-0.077 | ≤0.011 | ≥0.038 | 0.011-0.037 | ≤0.011 |

Abbreviations: OBS, oxidative balance score; BMI, body mass index.
